# Shared Leadership Improves Team Novelty: The Mechanism and Its Boundary Condition

**DOI:** 10.3389/fpsyg.2016.01964

**Published:** 2016-12-19

**Authors:** Xiaomin Sun, Yuan Jie, Yilu Wang, Gang Xue, Yan Liu

**Affiliations:** ^1^Beijing Key Lab of Applied Experimental Psychology, School of Psychology, Beijing Normal UniversityBeijing, China; ^2^School of Psychological and Cognitive Sciences, Peking UniversityBeijing, China; ^3^Department of Public Administration, Chinese Academy of GovernanceBeijing, China

**Keywords:** shared leadership, constructive controversy, team goal orientations, team creativity, learning goal orientation, performance goal orientation

## Abstract

Previous research has revealed the significant impact of shared leadership on team creativity, yet the mechanism underlying this relationship has rarely been investigated. The current research examined how shared leadership influenced team creativity (novelty and usefulness) across 3 studies using both long-term project teams and temporal task teams in the laboratory. The results showed that shared leadership enhanced the novelty dimension of team creativity by improving constructive controversy. Furthermore, team goal orientation moderated this effect. The indirect effect of constructive controversy holds for teams with learning goal orientation but not for those with performance goal orientation. Such patterns were not found in the usefulness dimension of team creativity.

## Introduction

In the process of globalization, work teams have come to be widely used in organizations to adapt to rapid market changes and foster innovation (Park et al., 2012). An increasing number of organizations rely on team creativity to boost their innovation (Miron-Spektor et al., 2012). By definition, team creativity refers to those team behaviors that produce both novel and useful solutions in a complex social context or work environment (Montag et al., 2012). Contrary to its popularity, relatively few studies have been conducted on team creativity compared with individual creativity (Lee et al., 2015). In this study, we explore the effects of shared leadership, team goal orientation, and constructive controversy on team creativity.

Previous research showed that leadership is essential to team effectiveness and creativity (Cohen and Bailey, 1997). Through the end of the twentieth century, most leadership theories and research focused on a single and formal leader. Recent years have witnessed the emergence of a broader view that sees leadership as an influencing process in organizations. According this view, there are two potential sources of leadership in an innovative team. One source is the vertical leader, and the other source is the team. Apart from the leadership functions performed by the formal leader in organizations, an increasing amount of attention is now devoted to examining how informal leadership functions of average team members contribute to team effectiveness (Friedrich et al., 2009).

Generated from the dynamic interaction among team members, shared leadership is an emerging character at the group level (Fletcher et al., 2003). Shared leadership is defined as “a dynamic, interactive influence process among individuals in groups for which the objective is to lead one another to the achievement of group or organizational goals or both (Pearce and Conger, 2002, p. 1).” Although, the positive influence of shared leadership on team effectiveness (Pearce and Sims, 2002; Muethel and Hoegl, 2013) and team performance (Mehra et al., 2006; Carson et al., 2007) has been largely confirmed by empirical research in various team contexts, research on the relationship between shared leadership and team creativity remains scant. The complexity and ambiguity facing innovative teams make it unrealistic for a single external leader to successfully perform all leading functions that are needed in an organization. Therefore, shared leadership can contribute to team creativity.

Much contemporary research on creativity has been guided by intrinsic motivation theory (Amabile, 1996; Oldham and Cummings, 1996). To understand how motivation influences team creativity, scholars have recently embraced goal orientation theory (Janssen and Van Yperen, 2004; Gong et al., 2009; Hirst et al., 2009). According to Bunderson and Sutcliffe (2003), there are two main orientations: (a) a learning goal orientation, with an emphasis on *developing* competence; and (b) a performance goal orientation, with an emphasis on *demonstrating* competence and avoiding failure. People with a learning goal orientation focus on learning, understanding, developing skills, and mastering information. People with a performance goal orientation focus on managing the impression that others have of their ability, attempting to create an impression of high ability and avoid creating an impression of low ability (Dweck, 1986). This is often done through comparison with the abilities of others (Nicholls, 1984). It is argued that a learning goal orientation will lead to more task-focused, adaptive, mastery-oriented behaviors, whereas a performance goal orientation will lead to more ego-focused, instrumental, and defensive behaviors (Dweck and Leggett, 1988). Based on previous research, team goal orientation is expected to moderate the association between shared leadership and team creativity such that learning goal orientation strengthens the association between shared leadership and team creativity.

Researchers have not yet agreed on how shared leadership influences team creativity. For innovative teams, their task and situation are mostly complex and non-routine. Accordingly, the teams often consist of members with different ideas and perspectives. How to address these differences or even conflicts will have a great impact on team creativity. Constructive controversy, a concept established by Johnson et al. (2006), refers to open discussions of opposite positions or opinions in pursuit of a common goal and collective benefits (Tjosvold and Yu, 2007). Constructive controversy is an important antecedent to positive team performance (Johnson et al., 1990) and team innovation (Chen et al., 2005). However, thus far, the constructive controversy has predominantly been treated as an input factor to team performance. Little is known about what factors are the important antecedents of constructive controversy. Shared leadership means members are more apt to collectively commit to their goal and cooperatively solve problems. Consequently, shared leadership in the present research is viewed as a predictor of constructive controversy.

The objective in this current study is to build on and extend prior research that has found support for shared leadership and team creativity by investigating additional variables present in team-based work structures. To this end, the present study examined the mediating role of constructive controversy and the moderating role of team goal orientation on the relationship between shared leadership and team creativity.

## Theoretical development

### Team creativity

Researchers have come to the consensus that teams act as “complex adaptive” systems (Ilgen et al., 2005, p. 519), which suggests that team creativity is affected not only by the intrinsic features of the team but also by the properties and behaviors of its members as well as by the properties of the overarching social context to which they belong. Using an “Input-Mediators-Outcome-Input” (IMOI) model as a conceptual guide, Cirella et al. (2014) captures the set of factors that have been considered important for team creativity in past studies into individual-level inputs, team-level inputs, team-level mediators (processes), team-level mediators (emergent states), and properties of macro-social system.

Diversity between individuals in a team was observed as one of the most important individual-level inputs influencing team creativity. However, studies on team diversity and creativity yielded mixed results. Diversity is defined as “the distribution of differences among the members of a unit with respect to a common attribute” (Harrison and Klein, 2007, p. 1200). Milliken and Martins (1996) have listed 14 different attributes potentially affecting group outcomes on which individuals differ (e.g., race/ethnic background, nationality, and gender). Researchers have argued that diversity may represent a “double-edged sword” (Milliken and Martins, 1996), as it may have both positive as well as negative consequences (Shore et al., 2009). Based on an information processing approach (Dahlin et al., 2005), diversity can be beneficial in more diverse teams because more heterogeneous knowledge and perspectives are available to teams, which would potentially increase team creativity. However, similarity-attraction paradigm (Byrne, 1997), social identity theory and self-categorization processes (Turner and Tajfel, 1986) argue that diversity can hinder group process by limiting common understandings and shared experiences or by creating such a divergence of ideas and styles that detrimental conflict can result (Shin et al., 2012). As team diversity may be a covariate of shared leadership and team creativity, it was (although the direction of its influence may not clearly be predictable) therefore included in this study as a control variable.

### Shared leadership and team creativity

Shared leadership has a potentially comprehensive influence on the contextual and structural characteristics of team innovation. This means multiple team members fulfill critical team leadership functions, collaboratively solve problems and collectively assume the responsibility for team outcomes. There is much evidence to support the positive effects of shared leadership on both team effectiveness (Muethel and Hoegl, 2013) and team performance (Mehra et al., 2006; Carson et al., 2007). Although the importance of shared leadership have been widely noted by scholars and practitioners, empirical examinations of the relationship between shared leadership and team creativity are very limited, among which, for example, Lee et al. (2015) showed that shared leadership positively contributed to team creativity in an e-learning environment. Wu and Cormican (2016) found that the density of a shared leadership network is positively related to team creativity in engineering design teams.

Past researchers have identified several environmental factors that are conducive to creativity: teams that are highly cooperative, teams that can self-determine procedures to perform tasks, organizations that have established a norm to actively share opinions, and so forth (Amabile, 2012). Shared leadership within a team precisely meets these conditions, such as autonomy for team members, support for cooperation among members with diverse expertise, and a team climate encouraging communication. Based on these studies, we proposed the following hypothesis:

**Hypothesis 1**. Shared leadership is positively related to team creativity.

Novelty and usefulness are frequently used as indicators of team creativity. Novelty means the degree to which the idea is unique from existing ideas. Usefulness indicates the degree of value offered by the idea within the organization and the broader domain in which it is embedded (Amabile, 1996; Berg, 2014). Previously, researchers have tended to assume that these two dimensions travel together in creative ideas (Oldham and Cummings, 1996; Shalley and Perry-Smith, 2001). However, increasing evidence suggests that novelty and usefulness are orthogonal dimensions (Ford and Gioia, 2000). Therefore, both dimensions were taken into consideration but examined separately in the current research.

### Shared leadership and vertical leadership

Shared leadership is a group process in which leadership is distributed among and stems from team members. Vertical leadership emphasizes that a certain managerial role outside (and above) the work team possesses formal authority over the team and is responsible for team process and consequences (Druskat and Wheeler, 2003). Although vertical leaders continue to play a significant role in developing and maintaining shared leadership, shared leadership should also play an important role in explaining team effectiveness, especially when team tasks are highly interdependent, highly complex, or require great levels of creativity (Pearce and Bruce, 2004). Pearce and Sims (2002), Pearce and Bruce (2004) and Ensley et al. (2006) found that shared leadership is a more useful predictor of team outcomes than vertical leadership—in change management and virtual and new venture teams, respectively. However, this is not necessarily to downplay the relative role of vertical leadership. As noted by Hoch (2013), “shared leadership is not mutually exclusive to other leadership forms and behaviors, but can be engaged in simultaneously with other approaches such as vertical leadership.”

To eliminate the possible effect of vertical leadership on team creativity, the current study did not assign vertical leadership in the teams, following other researchers in the field (e.g., Small and Rentsch, 2010). In this way, we could focus on the function of shared leadership on team creativity and on the underline mechanism of the function.

### The mediating role of constructive controversy

Controversy occurs when team members express their opposing ideas, opinions, conclusions, theories, and information that at least temporarily obstructs resolving an issue. Controversy within teams, when properly harnessed, could promote dialog and debate that stimulate innovation (Nonaka, 1990; Leonard and Straus, 1997; Leonard and Sensiper, 1998). Constructive controversy is defined as open-minded discussion of opposing perspectives for mutual benefit. The following are key components of constructive controversy: frankly expressing one's personal opinions, feeling uncertain instead of defensiveness about one's own positions, feeling eager to know and comprehend the opponent's arguments, viewing a situation or understanding of a concept from an alternate point-of-view, taking new and opposing information seriously, incorporating the arguments of the opponents into one's own ideas, and creating alternative solutions based on the more complete set of information (Tjosvold et al., 2009).

There are some facilitative interpersonal conditions that could promote constructive controversy, including cooperative goal interdependence, confirmation of personal competence, and collaborative influence (Tjosvold, 1985). Note that constructive controversy cannot be reduced to a sum of exchanged information; rather, ideas are continually built upon each other during the discussing process. It is through such mutual influence that people who hold different or even opposite views come to understand and appreciate one another. The result is idea generation, and ultimately, creative solutions. Shared leadership within a team is beneficial in that members become more involved in discussions and engage in deeper processing of ideas proposed by fellow members. Shared leadership also contributes to healthy criticism and comprehension of new information as well as to the integration of opposing ideas (Hoch, 2013). Hence, shared leadership promotes constructive controversy in teams.

In addition, constructive controversy has been found to enhance the quality of decision-making and team creativity. As diversity in ideas and opinions is almost unavoidable in decision-making or problem-solving within a team, an open and frank way to address conflicts is of great importance (Tjosvold, 1985). Constructive controversy guarantees a free and open discussion of diverse views. It also stimulates and activates team members' cognition and thinking (Tjosvold, 2008). Consequently, team members are more likely to promote creative decision-making, which leads to enhanced group-level creativity. Chen and Tjosvold (2002) reported an impact of constructive controversy on the innovation of teams. Alper et al. (2000) found that with more constructive controversy, the innovation of the team is higher. Open expression and frank communication of divergent opinions during the process alleviate possible tensions arising from dissents, facilitate idea integration among team members, give rise to transformational ideas and enhance team creativity (Alper et al., 2000; Chen et al., 2005). Based on this rationale, we expected that the increased constructive controversy triggered by shared leadership would enhance team creativity. Thus, we proposed the following hypothesis:

**Hypothesis 2**. Constructive controversy mediates the relationship between shared leadership and team creativity.

### The moderating role of team goal orientation

We proposed that shared leadership would influence team creativity through constructive controversy. However, this relationship may be moderated by other factors. Specifically, we zero in on the moderating role of team goal orientation.

Team goal orientation is defined as a consensus shared by team members about the goals of the team (Bunderson and Sutcliffe, 2003). As an emergent state, team-level goal orientation is subject to the influence of goal-relevant cues in the context, such as rewards and recognition (Maltarich et al., 2016), performance evaluation and organization policies (Druskat and Wheeler, 2003). For instance, in a social context where new ideas are valued, competency development is encouraged, and innovation is rewarded, a group climate in favor of learning orientation is more likely to form (Bunderson and Sutcliffe, 2003). By contrast, in an environment with competitive and persistent job evaluation and emphasis on performance-based rewards, a group climate favoring performance orientation is more likely to prevail (Dragoni, 2005).

For teams with differential goal orientations, the indirect effect of shared leadership on team creativity through constructive controversy may differ. Specifically, the constructive controversy triggered by shared leadership will promote team creativity when teams are led by learning goal orientation, which embodies group members' consensus of the goals of learning and competency development. According to Bunderson and Sutcliffe (2003), team-learning orientation determines the extent, range and intensity of learning behaviors in teams. Immersed in such a climate, the constructive controversy stimulated by shared leadership has beneficial effects: team members are encouraged to absorb instructive points from one another and persistently search for fresh ideas to deepen their understanding of the task and foster team competency. These efforts contribute to the promotion of team creativity in the objective sense.

In the case of performance orientation, the indirect effect of shared leadership on team creativity through constructive controversy may be weaker because such teams add more weight to the performance indicators than the task *per se*, tend to regard mistakes as evaluative threats (Martocchio and Frink, 1994), give up more easily when facing obstacles (Button et al., 1996), and contribute the least effort (Mangos and Steele-Johnson, 2001). As a result, the constructive controversy goes in the wrong direction (i.e., focusing on external performance appraisal instead of the ongoing task), and it is difficult to boost creative idea generation. Thus, we proposed the following hypothesis:

**Hypothesis 3**. Goal orientation moderates the indirect effect of constructive controversy on the relationship between shared leadership and team creativity. In particular, the indirect effect is stronger in learning goal orientated teams than performance goal-orientated teams.

### The present research

**Figure 1:**
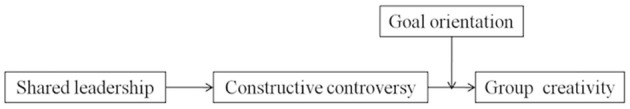
**Hypothesized model**.

Study 1 aimed to examine the positive impact of shared leadership on team creativity using the measurement of real-world task teams (Hypothesis 1). Study 2 further tapped into the mediating mechanism of constructive controversy in another wave of task teams (Hypothesis 2). In Study 3, we manipulated team goal orientation experimentally and tested its moderating effect on the relationship among shared leadership, constructive controversy and team creativity (Hypothesis 3). Taken together, we propose a moderated-mediation model (see Figure 1). The project was reviewed and approved by the Academic Ethics Committee of the School of Psychology at Beijing Normal University (approval number: 2015068) before being conducted.

This study contributes to the literature by providing theorists and researchers with a better understanding of the nature of shared leadership and its effect on team creativity. The current research is a reply to the appeal that more research attention should be devoted to examining the moderating and mediating variables of the shared leadership and team outcome (Pearce and Conger, 2002). Through our research, we deepen the understanding of how shared leadership increases team creativity and under what conditions this mechanism works better. Moreover, relatively few studies have been conducted on team level creativity (Lee et al., 2015). By considering the novelty and usefulness dimension of creativity separately, the current research is a valuable endeavor in exploring how different components of team creativity may respond differently to the shared leadership process of a team.

## Study 1

In this study, we investigated the influence of shared leadership on team creativity in long-term, real-world project teams. We expected that a higher level of shared leadership would predict greater team creativity.

### Methods

#### Participants and procedures

Fifty-five (40 females) students at a Chinese university in an undergraduate course participated in the study in exchange for course credit. The participants were randomly assigned to 19 groups, among which 17 groups have three persons each, and 2 groups have two persons each. The teams served as the basic unit for the course study and project task across the entire semester (18 weeks).

At the beginning of the semester, the participants were informed of this team research, and they all formally consented to participate. They were then randomly assigned to small teams. The task for the teams was to complete a research proposal for a self-selected project that aligned with the themes of the course. The proposal was required to be both original and practical. At the end of the semester, all teams submitted their research proposals, and every participant completed the measurement of shared leadership.

The reason why we choose to collect shared leadership data at the end of semester is because shared leadership is an emergent group property. It takes time to become a fully-fledged performance unit. As suggested by Perry et al. (1999, p. 43), “Shared leadership is a group process that requires time to develop, and its display is more likely in mature teams.” According to Carson et al. (2007), if shared leadership develops in a team, it will emerge over time through the interactions and mutual influence of its members. Therefore, if the conditions are suitable for leadership to emerge, a higher level shared leadership would be observed in the later stages of team development. A longitudinal analysis by Small and Rentsch (2010) revealed that shared leadership increased over time. In their study, students formed teams to complete a semester-long business simulation during which eight quarters of simulated business was conducted. Compared with data from Quarter 5, more shared leadership existed at Quarter 8, which was the conclusion of the simulation. Based on the above theoretical and empirical evidence, we decided to collect shared leadership data at the end of the semester, after the team had matured.

Moreover, each team also received a score for their daily performance in the course, which was the indicator of their academic achievement. The daily performance was based on 3 reflection papers over the course of the term. These were short papers written in response to course readings. The reflection papers were completed by the team, which discussed and collaborated together to compose the papers.

#### Measures

##### Shared leadership

The extent of shared leadership was assessed by 10 items adapted from Hiller (2002) (see Appendix). For example, “How often do team members share in developing solutions to problems?” The instrument measured shared leadership on a 7-point scale with responses ranging from 1 (never) to 7 (always). The scale was reliable in this study (α = 0.91).

##### Team creativity

Team creativity was assessed using the consensus assessment technique (CAT) (Amabile, 1982), which is a common practice in the field of creativity evaluation. Two graduate students familiar with the course yet blind to the purpose of the study independently rated the creativity (i.e., novelty and usefulness) of all the research proposals. Following O'Quin and Besemer (2006), each dimension has a total score of 10 points (1 = very low novelty/usefulness, 10 = very high novelty/usefulness). The consistency between the two raters was good, 0.87 for novelty and 0.81 for usefulness. Therefore, their ratings were averaged to determine team creativity (Duguid and Goncalo, 2015).

### Results

#### Preliminary analysis

To decide whether the individual-level data could be aggregated to the group level, we first calculated *r*_*wg*_ according to James et al. (1993). The average *r*_*wg*_ value of shared leadership was 0.97, which met the standard of 0.70 (James et al., 1984). Hence, the average score for individual shared leadership ratings in a group was a valid indicator of group-level shared leadership. Next, we calculated the ICC (1) (intraclass correlation) and ICC (2) (reliability of the group mean) to evaluate inter-group variance and intra-group consistency, respectively. For shared leadership, the ICC (1) value was 0.33, suggesting that 33% of the variance could be attributed to the group variable. The ICC (2) value was 0.60. Both ICC values were comparable to those found in previous group research (Carson et al., 2007; LeBreton and Senter, 2007; Liu et al., 2014; Song et al., 2015). Preliminary analysis verified the appropriateness of the data aggregation.

#### Shared leadership and team creativity

The descriptive statistics and correlations of shared leadership and team creativity (novelty and usefulness) are illustrated in Table 1. Correlation analysis showed that shared leadership was significantly positively associated with the novelty dimension of team creativity (*r* = 0.54, *p* < 0.01) but not correlated with the usefulness dimension (*r* = 0.36, *p* > 0.05). Given that the gender composition and academic achievement of the teams were significantly correlated with their creativity performance (as illustrated by Table 1), we further conducted a regression analysis controlling for these two variables. The results indicated that shared leadership positively predicted team novelty (β = 0.49, *p* < 0.05). Teams with higher levels of shared leadership completed research proposals that were more novel. Therefore, Hypothesis 1 was supported in terms of the novelty dimension of team creativity.

**Table 1:** **Correlations between shared leadership and team creativity (*N* = 19)**.

	***M***	***SD***	**1**	**2**	**3**	**4**
1. Gender composition	0.79	1.08				
2. Academic achievement	90.16	5.42	−0.75^**^			
3. Shared leadership	5.71	0.52	−0.51^*^	0.24		
4. Novelty	8.29	1.06	−0.53^*^	0.57^**^	0.54^*^	
5. Usefulness	8.47	1.10	−0.36	0.49^*^	0.36	0.39

* *p < 0.05*,

** *p < 0.01*.

## Study 2

Based on the findings of Study 1, in Study 2 we further examined the mechanism underlying this relationship by testing the mediating role of constructive controversy.

### Methods

#### Participants and procedures

Sixty-two (51 females) students at a Chinese university in an undergraduate course participated in the study in exchange for course credit. All participants gave their written informed consent on the first day of the course. The participants were randomly assigned to 21 groups, among which 20 groups have three persons each, and 1 group has two persons. The teams served as the basic unit for the course study and project tasks across the whole semester (18 weeks). The procedure was identical to the one for Study 1, except that we added the constructive controversy measure at the end of the semester.

#### Measures

##### Shared leadership

Shared leadership was measured by the 10-item questionnaire (α = 0.88) used in Study 1.

##### Team creativity

The evaluation of team creativity was identical to that of Study 1. Two graduate students independently rated the novelty and usefulness of all research proposals. The consistency between the two raters was good, 0.84 for novelty and 0.79 for usefulness. Their average ratings were used to determine team creativity.

##### Constructive controversy

Constructive controversy was measured by Li (2013)'s 6-item scale, which was adapted from Tjosvold's scale (Tjosvold, 2008) (see Appendix). One sample item was “Our team members expressed their own opinions directly to each other.” Participants indicated on a 7-point Likert scale (1 = strongly disagree, 7 = strongly agree) the extent of their agreement with 6 items that depict constructive controversy within the group. The reliability of the scale was good (α = 0.89).

### Results

#### Preliminary analysis

We calculated the *r*_*wg*_, ICC (1) and ICC (2) of shared leadership and constructive controversy measurement to determine whether individual-level data could be aggregated to group level. For shared leadership and constructive controversy, the average *r*_*wg*_ values were 0.97 and 0.96, the values of ICC (1) were 0.17 and 0.13, and the values of ICC (2) were 0.37 and 0.31, respectively, which justified the data aggregation (Carson et al., 2007; Liu et al., 2014).

#### Shared leadership and team creativity

Table 2 presented the descriptive statistics and correlations of shared leadership and team creativity (novelty and usefulness). Correlation analysis showed a positive relationship between shared leadership and novelty (*r* = 0.53, *p* < 0.01). The correlation between shared leadership and usefulness was not significant (*r* = 0.42, *p* > 0.05). A regression analysis with team gender composition as a control variable demonstrated that shared leadership positively predicted team novelty (β = 0.53, *p* < 0.05). In other words, team creativity improved as the level of shared leadership increased. The result is consistent with those of Study 1, which support Hypothesis 1 on novelty dimension of team creativity.

**Table 2:** **Means, standard deviations, and correlations of the main variables (*N* = 21)**.

	***M***	***SD***	**1**	**2**	**3**	**4**	**5**
1. Gender composition	0.55	–					
2. Academic achievement	88.05	4.70	−0.41^*^				
3. Shared leadership	5.79	0.42	−0.16	0.04			
4. Constructive controversy	6.18	0.43	0.07	−0.32	0.59^**^		
5. Novelty	5.24	0.85	−0.40^*^	0.01	0.53^**^	0.68^**^	
6. Usefulness	5.19	0.96	−0.71^**^	0.32	0.42	0.16	0.47^*^

* *p < 0.05*,

** *p < 0.01*.

#### Mediation analysis

As shown in Table 2, novelty was significantly related to shared leadership (*r* = 0.53, *p* < 0.01) and constructive controversy (*r* = 0.68, *p* < 0.01), whereas usefulness was associated with neither shared leadership (*r* = 0.42, *p* > 0.05) nor constructive controversy (*r* = 0.16, *p* > 0.05). Controlling for the effect of team gender composition, regression analysis showed that both shared leadership (β = 0.48, *p* < 0.05) and constructive controversy (β = 0.69, *p* < 0.001) significantly predicted the novelty dimension of team creativity. Moreover, the predictive effect of shared leadership on constructive controversy was also significant (β = 0.63, *p* < 0.001).

**Figure 2:**
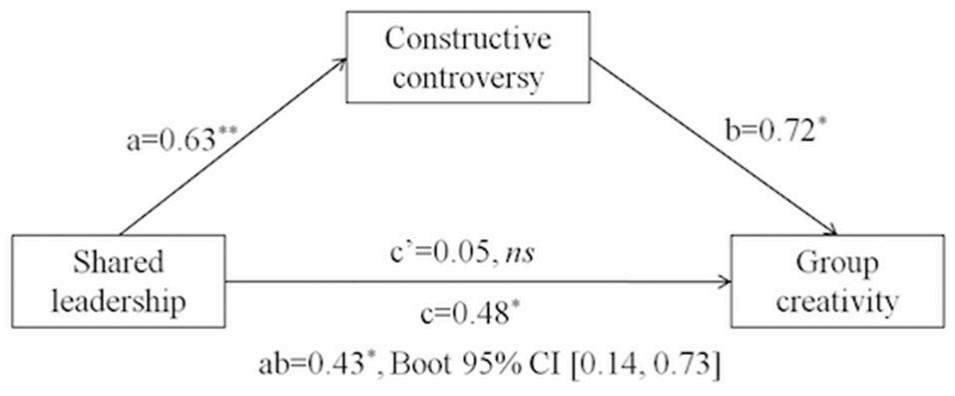
**Mediating model in Study 2 with controls including gender composition and academic achievement**.

We tested the mediating role of constructive controversy using the BOOTSTRAP test (Hayes, 2013). The software used for analysis included SPSS 22.0 and Mplus 7.0. A bootstrapping analysis controlling for gender composition and academic achievement revealed that constructive controversy mediated the relationship between shared leadership and novelty (see Figure 2), ab = 0.43, *p* < 0.05, 95% CI = [0.14, 0.73]. The mediation accounted for 89.58% (ab/c = 0.43/0.48) of the total effect. The direct effect was insignificant, c' = 0.05, *p* > 0.05. The results partly supported Hypothesis 2 in that the mediating role was confirmed for novelty dimension but not for usefulness dimension.

## Study 3

In Study 3, we further examined the boundary conditions of the established shared leadership–team novelty relationship and the mediating role of constructive controversy. In particular, we paid attention to the goal orientations of task teams.

### Methods

#### Participants and design

One hundred and eight undergraduate students (*M*_age_ = 21.73, *SD*_age_ = 2.14) were recruited through advertisements on campus. Given the extremely high proportion of female students at the university where the current study was conducted, we included only female participants to avoid potential confusion arising from different gender compositions. All participants gave their written informed consent prior to the experiment and were informed of their right to abort the experiment at any time. The participants were randomly assigned to teams of three members each, and teams were randomly assigned to one of two conditions. We had 19 teams (57 participants) in the learning orientation condition and 17 teams (51 participants) in the performance orientation condition. After the experiment, each person received 25 RMB for participating.

#### Procedure and task

Upon arrival at the laboratory, participants were informed that they would join teams with another two partners to perform a team task, and they then signed the consent form.

Goal orientation was manipulated through instructions, which were adapted from Miron-Spektor and Beenen (2015). In the learning orientation condition, the instructions read as follows: “Your team is going to complete a task on product design. The purpose of the task is to improve the creative skills of your team. We would like you to learn from and communicate with fellow members during the interaction. Please focus on the development and learning of team creative skills. Don't worry about making mistakes. Mistakes are a natural part of the learning process.” The instructions for the performance orientation condition were as follows: “Your team is going to complete a task on product design. The purpose of the task is to evaluate the creative level of your team. We would like you to focus on displaying your team's creativity and showing that you are more creative than other teams by generating the most and best ideas for the product.” After the instruction, as a manipulation check, the learning and performance goal mindset of each participant was measured.

The team creativity task aimed to generate both novel and useful plans to improve the functions of shopping carts, a task adapted from Li (2013). Each team was given 20 min for discussion before submitting their ideas. Finally, the participants completed measures of shared leadership and constructive controversy.

#### Measures

##### Shared leadership

We used social network analysis (SNA) (Mayo and Pastor, 2002) to assess the level of shared leadership in this study. Each team member responded to the question “To what extent does your team rely on this person's leadership” on a 7-point frequency scale (1 = not at all, 7 = to a very great extent) following Carson et al. (2007). The targets of evaluation were fellow members on the team, such that a leadership rating was attributed to each individual by every other team member. These data were analyzed following a social network approach by using density according to Carson et al. (2007). Shared leadership density is a measure of the total amount of leadership displayed by team members as perceived by others on a team. It precisely captured the core characteristic of shared leadership, namely, the extent to which leading behaviors were distributed among all team members. Previous research also lent support to the validity of the density measurement (Ishikawa, 2012; Liu et al., 2014).

##### Team creativity

Two independent raters that were blind to the purpose and conditions of the study rated the novelty and usefulness on a 7-point scale (1 = low, 7 = high) of all the teams' plans. By novelty, we mean that the plan was original and unique. Usefulness indicates whether the plan is practical with pragmatic values. The ratings of the two raters were highly correlated, 0.80 for novelty and 0.83 for usefulness.

##### Constructive controversy

We used the same constructive controversy measure as Study 2. Cronbach's α for the scale was 0.85.

##### Team goal orientation

Following Miron-Spektor and Beenen (2015), we adapted the goal orientation measures by Barron and Harackiewicz (2001) based on our experimental situations. Learning orientation was measured by 4 items (α = 0.93) such as, “We wanted to improve and develop our creative skills on the task.” Performance orientation was measured by 4 items (α = 0.94) such as, “The goal of our team in this task was to display our creativity to others by making the best product.” Participants reported (on a 7-point scale ranging from 1 = strongly disagree, to 7 = strongly agree) the extent to which they agree with these statements.

### Results

#### Validation of data aggregation

As in Studies 1 and 2, we calculated *r*_*wg*_, ICC (1) and ICC (2) to determine the legitimacy of data aggregation. The average values of *r*_*wg*_, ICC (1) and ICC (2) were 0.91, 0.31, and 0.48 for shared leadership, 0.97, 0.12, and 0.29 for constructive controversy, 0.89, 0.62, and 0.83 for learning orientation, and 0.94, 0.69, and 0.87 for performance orientation. Thus, the calculations of shared leadership, constructive controversy, and goal orientation at the team level were deemed appropriate.

#### Manipulation check

We first conducted an independent samples *t*-test on the individual level to validate the goal orientation manipulations. As expected, learning orientation ratings were higher in the learning orientation condition (*M* = 6.18, *SD* = 0.64) than in the performance orientation condition [*M* = 3.87, *SD* = 1.54, *t*_(106)_ = 10.35, *p* < 0.001]. Performance orientation ratings were higher in the performance orientation condition (*M* = 6.16, *SD* = 0.86) than in the learning orientation condition [*M* = 3.66, *SD* = 1.36, *t*_(106)_ = 11.29, *p* < 0.001]. Therefore, our manipulation was successful at the individual level. We further tested the between-group differences of goal orientation ratings at the group level. As expected, team learning orientation ratings were higher in the learning orientation condition (*M* = 6.18, *SD* = 0.39) than in the performance orientation condition [*M* = 3.87, *SD* = 1.13, *t*_(34)_ = 8.04, *p* < 0.001]. Team performance orientation ratings were higher in the performance orientation condition (*M* = 6.17, *SD* = 0.77) than in the learning orientation condition [*M* = 3.66, *SD* = 0.93, *t*_(34)_ = 8.75, *p* < 0.001]. These results showed that our manipulation was effective in activating learning and performance goals.

#### Preliminary analysis

Table 3 displays the means, standard deviations and correlations among the main measures. Shared leadership was positively associated with constructive controversy (*r* = 0.56, *p* < 0.05) and novelty (*r* = 0.68, *p* < 0.05) but not with usefulness (*r* = 0.20, *p* > 0.05). Constructive controversy was positively related to novelty (*r* = 0.58, *p* < 0.05), but its correlation with usefulness was not significant (*r* = 0.27, *p* > 0.05). Therefore, the usefulness dimension of team creativity was not included in the following analysis.

**Table 3:** **Descriptive statistics and correlations among the main measures (*N* = 36)**.

	***M***	***SD***	**1**	**2**	**3**	**4**
1. Shared leadership (SNA)	2.83	0.22				
2. Constructive controversy	6.48	0.27	0.56^**^			
3. Novelty	5.89	0.46	0.68^**^	0.58^**^		
4. Usefulness	5.01	0.78	0.20	0.27	0.30	
5. Goal orientation^a^	0.47	0.51	−0.11	0.07	−0.20	−0.09

^*^ p < 0.05,

** *p < 0.01*.

a *Goal orientation is a dummy variable, with learning orientation = 0 and performance orientation = 1*.

#### Hypothesis testing

We tested the proposed moderated-mediation model using the BOOTSTRAP test (Hayes, 2013). The mediator (constructive controversy) and moderator (goal orientation) were centered before creating the interaction item. The results (see Table 4) showed that the interaction of goal orientation and constructive controversy significantly predicted the novelty dimension of team creativity (*t* = −3.33, β = −0.40, *p* < 0.01). Simple slope analysis results further suggested that team goal orientation moderated the mediating role of constructive controversy in the relationship between shared leadership and team novelty. As illustrated by Table 4, in the learning orientation condition, the indirect effect of constructive controversy was significant, *p* < 0.01, Boot 95% CI = [0.19, 0.97]; conversely, the indirect effect was not significant in the performance orientation condition, *p* > 0.05, Boot 95% CI = [−0.96, 0.19]. Hypothesis 3 was partly supported.

Table 4**Moderated-mediation analysis**.

**Table:** 

**Predictor**	**Team creativity (novelty)**		
	**β**	***SE***	***t***
Independent variable			
Shared leadership (SNA)	0.47	0.13	3.73^**^
Mediator			
Constructive controversy	0.54	0.14	3.80^**^
Moderator			
Goal orientation (learning/performance)	−0.15	0.21	−1.46
Interaction			
Constructive controversy ^*^ goal orientation	−0.40	0.24	−3.33^**^
*R^2^*	0.67		
*F*	11.08^**^

**Table:** 

**Moderated-mediation analysis**	**Moderator**	**level**	**Indirect effect**	***SE***	**Boot 95% CI**
	Goal orientation (LO/PO)	LO	0.65	0.15	[0.19, 0.97]
		PO	−0.38	0.22	[−0.96, 0.19]

** *p < 0.01*.

*β is the standardized regression coefficient. The number of bootstrap samples was set at 1000*.

## General discussion

In the era of globalization with fierce competition, creativity is the soul of organizations. Teams gradually become the unit of innovation in place of individuals with a specialized division of labor and rapid development of science and technology. An urgent issue emerges for managerial practice and organizational behavior research with respect to how to promote team creativity.

The current research adopted a perspective based on the leadership style of the team. Across three studies, we found that the level of shared leadership positively predicted the novelty dimension of team creativity. Moreover, constructive controversy mediated the relationship. We also examined one boundary condition for this effect. When the teams were learning-oriented, the constructive controversy stimulated by shared leadership promoted team creativity; however, when the teams were performance-oriented, the indirect effect was not significant.

### Theoretical implications

Firstly, researchers have not yet agreed on how shared leadership influences team innovation. Our finding that shared leadership enhanced team creativity by facilitating constructive controversy provides an explanation of the underlying mechanism. Song et al. (2015) investigated how shared leadership influences team performance and team creativity, respectively. In their study, shared leadership significantly influenced team performance through the cognitive mechanism of information exchange. However, the anticipated mediating role of information exchange between shared leadership and team creativity was not confirmed. We contend that a clashing and merging process of ideas and knowledge among members beyond mere information exchange is necessary for achieving a high level of team creativity. This core characteristic of team creativity is captured by the concept of constructive controversy. Constructive controversy emphasizes that team members openly discuss their opposite opinions, focus on cooperation and interdependence in pursuing collective goals, confirm the positions of their opponents based on their own evidence, and give more attention to the generation of new ideas based on divergent information and perspectives instead of being anxious to win the debate (Tjosvold, 1985). What makes constructive controversy different from information sharing is that during the process there is not only information exchange but also mutual inspiration and support of different perspectives. It is through such a thorough mutual understanding and appreciation process that new ideas and solutions are generated as the result of a collective endeavor.

Secondly, we noted a boundary condition for the established relationship between shared leadership and team creativity. Although shared leadership can spur the integration of different ideas and perspectives in a team (i.e., constructive controversy), its positive effects may be suppressed when the team is performance goal oriented. This suppression occurs probably because when the team places too much emphasis on performance, members become more concerned with the evaluations of others, longing for appreciation and avoiding failure. Conversely, learning goal-oriented teams focus on developing competency, engage in more active learning behaviors, and strive to generate more new solutions, thus ensuring the positive role of shared leadership and constructive controversy.

Thirdly, we found that the mediation mechanism between shared leadership and team creativity and its boundary condition hold only for the novelty component of creativity, not for the usefulness dimension. This finding is consistent with the existing literature. More and more scholars recognized that it may be rare for ideas to be observed as high in both novelty and usefulness (Rietzschel et al., 2010; Mueller et al., 2012) and suggested that novelty and usefulness probably are motivated by different psychological processes (Rietzschel et al., 2010; Grant and Berry, 2011; Mueller et al., 2012; Berg, 2014). For example, individuals generate useful solutions under conditions when they are experiencing cognitive closure (Miron-Spektor and Beenen, 2015), or when they are anxious to reduce uncertainty by drawing on existing practices (Janssen and Van Yperen, 2004; Mueller et al., 2012). In contrast, the generation of novel ideas is most likely when individuals are intrinsically motivated (Grant and Berry, 2011), cognitively flexible (Miron-Spektor and Beenen, 2015), eager to learn new domains, and feel safe to take risks (Hirst et al., 2009). These conditions are exactly what shared leadership and learning goal orientation could jointly offer. Past research on shared leadership and team creativity has tended to examine novelty and usefulness together in one overall creativity construct (Oldham and Cummings, 1996; Shalley and Perry-Smith, 2001). By exploring novelty and usefulness separately, the current study deepens our understanding as to how each aspect of team creativity benefits from shared leadership. Future research could also explore mechanisms under which the usefulness dimension of team creativity could be improved.

### Practical implications

Team creativity is of great significance for the success of organizations. The findings of our study thus may have useful implications to management practitioners.

Firstly, team creativity could be promoted by advocating shared leadership in teams. Demands for team creativity, ranging from designing products to innovating working ideas, are ubiquitous in contemporary organizations. If we could create certain conditions to facilitate the development of shared leadership, team novelty and competitiveness of the organization would improve accordingly. As suggested by Pearce and Bruce (2004), training systems can be used to develop the shared leadership skills of both vertical leaders and team members; reward systems can be used to promote shared leadership in teams; and cultural systems can be used to emphasize the significance of shared leadership.

Secondly, as constructive controversy plays a critical role in the effect of shared leadership on team creativity, a group atmosphere of freely expressed dissent is worthy of advocacy. To achieve this state, managers could emphasize cooperative goals in teams. By doing so, members would be more motivated to take into account the rational parts of other's thoughts, seeking verification and elaboration of different opinions rather than antagonism.

Thirdly, managers should pay close attention to team goal orientations and create a benign climate for learning. For example, situational cues, group norms, organization policies, and cultural channels can be employed to convey the value of learning treasured by the organization. Only in such conditions can the advantage of shared leadership transfer to team creativity through active interactions. Several managerial behaviors are likely to foster team learning goal orientation. First, individual differences in learning orientation should be considered when selecting team members. However, putting high learning-oriented individuals on the team is not enough. What is even more important is for managers to provide support and encouragement to alleviate employee fear and anxiety that may arise from the uncertainty of creative endeavors. When teams experience failure, managers could provide educational coaching emphasis on what precious lessons the teams have learned during the process and what could have been done to make the result better. This support and coaching should nurture a the learning orientation of the team and boost team novelty, as suggested by the current research.

### Limitations and future directions

Several limitations of the current research should be addressed in future studies.

Firstly, as mentioned earlier, team diversity is an important individual-level input influencing team creativity, although past research on team diversity has yielded mixed results (Williams and O'Reilly, 1998; Jackson and Joshi, 2004; Harrison and Humphrey, 2010). Shin et al. (2012) suggest that diversity should have conceptual relevance to the outcome variables, that is, be task-related. To focus on the mechanism of how shared leadership influences team creativity, the current study did not test the impact of team diversity. Future research could take task-related team diversity into consideration and test how team diversity influences the relationship between shared leadership and team creativity. We propose that when task-related team diversity is high, shared leadership would have a stronger influence on team creativity because teams could benefit more from shared leadership when the competencies of their members differ from each other.

Secondly, to highlight the important function of shared leadership on team creativity does not mean that vertical leadership is dispensable. The results of this study provide robust evidence for the value of shared leadership. Findings in this regard can not only help establish the legitimacy of shared leadership research but also provide fruitful future research directions on the potential interplay between shared and vertical leadership. Future studies could explore how one utilizes both vertical and shared leadership to leverage the capabilities of knowledge workers in innovative teams.

Thirdly, the current study was conducted in China. The generalizability of the research results to other cultures needs further evidence. Some cultural dimensions may shape the relationship between shared leadership and team creativity. Take power distance, for example. China is a high-power distance culture. When shared leadership is advocated in teams embedded in a high-power distance culture, team creativity may benefit more, compared with those teams in low-power distance settings. The reason is because vertical leadership is a rather dominant role in high-power distance culture, and team members normally accept the authority of vertical leadership without questioning. When shared leadership was introduced in such teams, the wisdom and valuable input of team members could be released and team creativity could be boosted substantially. However, things may be different in a low-power distance culture. Further research could be conducted in different cultures to testify to the findings of the current research. Additionally, collecting data from teams in organizations will also contribute to the external validity of the findings of the current study.

Another limitation is that we manipulated team's performance orientation following the instruction of Miron-Spektor and Beenen (2015), which is widely used in the field of team research. The instruction introduced a competitive framework that could possibly generate anxiety or tension in team members and therefore might have a negative impact on the level of team creativity. Future research could make additional effort to manipulate performance orientation without inducing potential confounding affect (e.g., anxiety) in teams.

Finally, in our research, we manipulated two types of team goal orientations (i.e., learning and performance orientation), which have also been confirmed in the extant literature at the group level. Theories and practice of goal orientation at the individual level, however, provided a distinction between 3 goal orientations, namely, learning, performance-prove and performance-avoid orientations (Harackiewicz et al., 2002). Despite their common concern with the task, individuals with a performance-prove orientation have a strong desire to prove their ability, whereas those with a performance-avoid orientation mainly think about avoiding failure (Elliot and Church, 1997). A distinction between the two performance orientations has been proposed yet has rarely been studied empirically (Druskat and Wheeler, 2003). We believe that the three-dimensional frame of goal orientation provides another avenue for future researchers to deepen their understanding of its moderating mechanism in the constructive controversy-mediated relationship between shared leadership and team creativity.

## Author contributions

Conceived and designed the experiments: XS and YJ. Program the task: YJ, XS, and GX. Performed the experiments: YJ and YL. Analyzed the data: YJ. Wrote the paper: XS, YJ, and YW.

## Funding

This research is funded by the National Natural Science Foundation of China (Grant No. 71101012), the Fundamental Research Funds for the Central Universities, and the State Scholarship Fund from the China Scholarship Council (201406045033).

### Conflict of interest statement

The authors declare that the research was conducted in the absence of any commercial or financial relationships that could be construed as a potential conflict of interest.
