# Supplementary material for: Shared Leadership Improves Team Novelty: The Mechanism and Its Boundary Condition
Source: Front Psychol. 2016 Dec 19;7:1964. doi: 10.3389/fpsyg.2016.01964 (PMC5165241; doi:10.3389/fpsyg.2016.01964)
Supplement: Supplementary file 1 [file DataSheet1.docx]

Appendix

Shared leadership scale

How often do team members share in:

1. setting our team’s goals.
2. providing helpful input about our team’s work-related plans.
3. deciding on the best course of action when a problem arises.
4. providing support to team members who need help.
5. developing solutions to problems.
6. allocating team resources according to our team’s priorities.
7. identifying problems before they arise.
8. fostering a cohesive team atmosphere.
9. diagnosing problems quickly.
10. instructing poor performers on how to improve.

Constructive controversy scale

1. Our group members expressed own opinions directly to each other.
2. Our group members attended to other’s ideas.
3. Our group members tried to understand other members in the discussion.
4. Our group members tried to integrate other members’ opinions.
5. Our group members showed mutual respect despite disagreements.
6. Our group members worked for decisions that we agreed upon.
